# A continuously updated, geospatially rectified database of utility-scale wind turbines in the United States

**DOI:** 10.1038/s41597-020-0353-6

**Published:** 2020-01-13

**Authors:** Joseph T. Rand, Louisa A. Kramer, Christopher P. Garrity, Ben D. Hoen, Jay E. Diffendorfer, Hannah E. Hunt, Michael Spears

**Affiliations:** 1 Lawrence Berkeley National Laboratory, Electricity Markets and Policy Group, Berkeley, CA 94720 USA; 2 US Geological Survey, Geosciences and Environmental Change Science Center, Denver, CO 80225 USA; 3 US Geological Survey, Eastern Energy Resources Science Center, Reston, VA 20192 USA; 4 American Wind Energy Association, Washington, DC 20005 USA

**Keywords:** Power stations, Energy and society, Energy and society, Wind energy, Energy modelling

## Abstract

Over 60,000 utility-scale wind turbines are installed in the United States as of October, 2019, representing over 97 gigawatts of electric power capacity; US wind turbine installations continue to grow at a rapid pace. Yet, until April 2018, no publicly-available, regularly updated data source existed to describe those turbines and their locations. Under a cooperative research and development agreement, analysts from three organizations collaborated to develop and release the United States Wind Turbine Database (USWTDB) - a publicly available, continuously updated, spatially rectified data source of locations and attributes of utility-scale wind turbines in the United States. Technical specifications and wind facility data, incorporated from five sources, undergo rigorous quality control. The location of each turbine is visually verified using high-resolution aerial imagery. The quarterly-updated data are available in a variety of formats, including an interactive web application, comma-separated values (CSV), shapefile, and application programming interface (API). The data are used widely by academic researchers, engineers and developers from wind energy companies, government agencies, planners, educators, and the general public.

## Abstract (summary)

**Table:** 

Measurement(s)	geographic location • instrument attribute
Technology Type(s)	digital curation
Sample Characteristic - Environment	anthropogenic environment
Sample Characteristic - Location	United States of America

Machine-accessible metadata file describing the reported data: 10.6084/m9.figshare.11441310

## Background & Summary

In 2015, the United States Geological Survey (USGS) released a publicly-available dataset of utility-scale wind turbine (WT) locations for the United States to March 2014^1,2^. Since then, over 18,700 new WTs have been installed in the US, representing over 40 gigawatts (GW) of installed capacity, and over 4,900 additional WTs (representing an estimated 0.7 GW) have reached the end of their useful life and been dismantled and removed through the third quarter of 2019^3^. USGS did not update the wind turbine data after 2015 and it quickly became outdated.

Independently, researchers from Lawrence Berkeley National Laboratory (LBNL) and the American Wind Energy Association (AWEA) had been updating and maintaining separate WT datasets^4,5^, but those datasets were not publicly available. The Federal Aviation Administration (FAA) also maintains two datasets that include WT locations, but these have little information on WT characteristics, unvalidated geospatial accuracy, limited screening for dismantled WTs, and few smaller WTs less than 61 meters (200 feet) in total height (the minimum structure height for which FAA is tasked to maintain data)^6,7^.

In 2016, LBNL with support from the US Department of Energy (DOE), USGS, and AWEA began collaborating under a cooperative research and development agreement (CRADA) to develop the US Wind Turbine Database (USWTDB). The goal was to create a unified, continuously updated, publicly-available database that would be more comprehensive and accurate than the WT datasets maintained by each organization separately. Federal agencies began using these combined data in April 2017, and in April 2018 the data were released to the public via an online portal^8^ (see https://eerscmap.usgs.gov/uswtdb/) and as a downloadable database^3^.

The USWTDB is the most comprehensive, accurate, and complete description of a nation’s wind energy infrastructure publicly available anywhere in the world. The corresponding web-based user interface makes the data highly accessible, intuitive, and usable by the public and researchers. By the end of October 2019, the web-viewer had been used over 2,800,000 times, and the raw data in CSV, Shapefile, and Geo-JSON formats downloaded over 8,500 times since the release of the database in April 2018.

Based on website analytics and the feedback mechanism built into the web-viewer, the authors know that the web-viewer and data products are used by: developers, technicians, and engineers from wind energy companies; grid system operators; academic researchers; the US Department of Defense; federal, state, and county government agencies; natural resource managers; educators; and the general public, to name a few.

Accurate and up-to-date spatial information about WTs in the US is critical for a range of research applications, such as: assessments of climate and health benefits of wind power^9^; analyses of electric grid impacts and requirements related to high penetrations of renewable energy^10,11^; estimates of capacity factors, power densities^12^, wake effects^13^ and land requirements^14^ of wind energy; impacts of WTs on local climate and surface temperature^15^; mitigation of radar interference from WTs^16^; assessments of bird and bat collisions with WTs^17^; estimates of the wind resource quality of the US wind fleet^18^; analyses of public acceptance^19–21^, sound annoyance^22,23^, rural land use preferences^24^, and changes to property values associated with WTs^25,26^; and geospatial analyses of renewable energy technical potential^27,28^.

As of October 2019, the USWTDB contains 60,576 utility-scale (typically ≥100 kilowatts (kW), though very old WTs may be smaller and still considered utility-scale) WTs spread across 43 US states, Guam, and Puerto Rico. Upon final submission of this article, the most recent WTs added to the USWTDB became operational as recently as the second quarter of 2019, with a few from the third quarter 2019.

The geodetic location (latitude and longitude in North American Datum of 1983 (NAD83)) is provided for each WT. The WTs’ locations have been visually verified using high-resolution satellite imagery and given a “location confidence” score based on a set of well-defined criteria, as outlined in the methods section below. WT locations are not verified in the field.

In addition to location, the USWTDB provides a number of attributes, including: unique identifiers (IDs) to link to FAA datasets and the 2014 USGS dataset; state and county; manufacturer; model; rated power capacity; hub height; rotor diameter; rotor swept area; and total height. In addition, each WT is grouped into a wind project and project-level attributes are also provided, including: project name; year online; total number of WTs; and total project power capacity.

The USWTDB is updated on a quarterly basis. Each release updates the database with newly installed WTs, removes WTs that have been identified as dismantled, and applies other changes based on ongoing quality-control (QC). A written memo, data codebook, Federal Geographic Data Committee (FGDC) compliant metadata, and a changelog also accompany each quarterly update.

## Methods

### Data sources

The USWTDB incorporates WT location and attribute data from 5 primary sources, shown in Table 1.

**Table 1:** Primary data sources used in the creation of the USWTDB. *For USWTDB V 2.2.

Source	Acronym	Release Date*	Regularly Updated? (Frequency)	Citation Number
2014 USGS Turbine Dataset	USGS	May 2015	No	2
LBNL Wind Technologies Market Report Turbine Dataset	LBNL	December 2016	No	4
AWEA WindIQ Turbine Dataset	AWEA	July 2019	Yes (Quarterly)	5
FAA Digital Obstacle File	DOF	July 2019	Yes (56 Days)	6
FAA Obstruction Evaluation/Airport Airspace Analysis	OE-AAA	July 2019	Yes (Weekly)	7

The AWEA, FAA DOF, and FAA OE-AAA datasets are regularly updated, while the USGS and LBNL WT datasets have been superseded by the USWTDB and are no longer maintained. LBNL still maintains and regularly updates a tabular (non-spatial) dataset of wind energy *projects* (wind farms) but not of individual WTs. USGS and LBNL WT datasets were used as the starting point for the initial USWTDB dataset.

The AWEA WindIQ dataset is secured behind a paywall and accessible to AWEA members only. However, through the CRADA with LBNL, USGS, and AWEA, they agreed to share WT locations, some turbine attributes, and wind project characteristics publicly through the USWTDB. The WindIQ dataset is updated quarterly by AWEA.

The FAA datasets contain *all* potential aviation obstructions (flight obstacles) in the United States, not just wind WTs. The FAA updates and releases a new DOF every 56 days, and the OE-AAA weekly. To compile WT-only datasets, USWTDB analysts download the DOF and OE-AAA files, filter them for WTs, clean them of extraneous fields, and add a few derivative fields (e.g., a unique ID without dashes). The analysts process and store the weekly FAA files, but only the most recent versions are incorporated into the USWTDB during the quarterly update process.

### Merging data

**Fig. 1:**
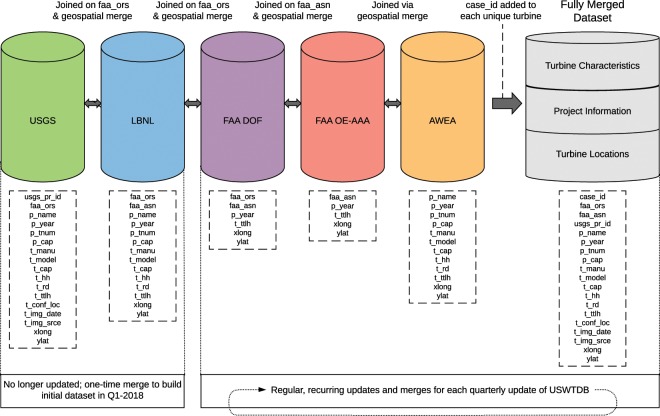
Overview of source data merging processes.

Analysts merge the distinct data sources into the USWTDB in a manner that keeps all relevant individual WT characteristics from each dataset. This results in a dataset with more depth (i.e., more attribute fields) and breadth (i.e., more WT records) compared with any single-source dataset. Two merging methods are used: First, available tables are merged using unique IDs shared between two datasets (full outer join). Second, for any records lacking a shared unique ID, a geospatial matching algorithm is employed, which uses the WTs’ latitude, longitude, and attributes to determine matches. As the datasets are combined and merged, each WT is assigned a new unique, stable ID (case_id) for subsequent tracking, merging, and analysis. The merging process is described in detail in the two subsections below, and a graphical outline of the merging processes is shown in Fig. 1.

#### ID merging via full outer join

Some unique WT ID fields are shared across multiple source datasets. The USGS, LBNL, and DOF datasets contain the FAA Obstacle Repository System (ORS) number, while the LBNL, DOF, and OE-AAA datasets contain the FAA Aeronautical Study Number (ASN) for many WTs. The AWEA dataset does not have any unique identifying fields in common with the other datasets.

To compile the initial combined dataset, the 2015 USGS dataset was merged with the 2016 LBNL dataset using the ORS number and a full outer join (thus retaining both matching and non-matching records from both tables). Attribute fields from USGS and LBNL were kept for each matching WT (see Table 2 for variables retained from each source dataset). In cases where USGS and LBNL attribute data conflicted in a matching WT, LBNL data took precedence (as described in the “Wind Turbine and Project Attributes” section, below). The non-matching WTs from both datasets were kept and subsequently checked using geospatial matching (described below) to ensure that duplicate WTs were not retained.

**Table 2:** USWTDB variable sources.

Characteristic	Variable Name	USGS	LBNL	AWEA	DOF	OE-AAA	Added by Analyst	Used for t_conf_atr
Unique ID	case_id						•	
FAA ORS Number	faa_ors	•	•		•			
FAA ASN Number	faa_asn		•		•	•		
USGS Prior ID	usgs_pr_id	•						
Turbine state	t_state						•	
Turbine county	t_county						•	
Turbine FIPS code	t_fips						•	
Project name	p_name	•	•	•			•	•
Project online year	p_year	•	•	•	•	•		•
Project num. turbines	p_tnum	•	•				•	
Project capacity	p_cap	•	•				•	•
Turbine manufacturer	t_manu	•	•	•				•
Turbine model	t_model	•	•	•				•
Turbine capacity	t_cap	•	•	•				•
Hub height	t_hh	•	•	•				•
Rotor diameter	t_rd	•	•	•				•
Rotor swept area	t_rsa						•	
Total height	t_ttlh	•	•	•	•	•		•
Attribute confidence	t_conf_atr						•	
Location confidence	t_conf_loc	•					•	
Image date	t_img_date	•					•	
Image source	t_img_srce	•					•	
Latitude	ylat	•	•	•	•	•		
Longitude	xlong	•	•	•	•	•		

Variable names are shown as they appear in the USWTDB, not in source datasets.

The initial merge of the USGS and LBNL dataset described above was conducted only once, because the USGS and LBNL datasets are no longer updated. The process described below is conducted quarterly to maintain and update the USWTDB.

Quarterly updates of the previous dataset proceed as follows: The updated DOF is merged into the previous dataset, joined by ORS number. These merged WTs have several fields that are retained from the DOF (see Table 2). The non-matching WTs are kept for subsequent geospatial matching and visual verification.

Because the LBNL and the DOF datasets both contain the FAA ASN number, analysts merge the OE-AAA data into the combined dataset, joining on ASN. Several fields are retained from the OE-AAA (see Table 2). The non-matching WTs are kept for subsequent geospatial matching and visual verification.

#### Merging via geospatial matching

The outer join merging described above leaves a number of unmatched WTs from each source dataset. A geospatial matching technique is used to determine which of those unmatched WTs should, in fact, be merged together.

In addition, the AWEA dataset is merged into the combined dataset using similar geospatial matching techniques, because it contains no WT ID that is shared with other source datasets. To determine a match, the geodetic distance between WTs and WT attributes are compared. Geodetic distance between WTs is calculated between a pair of X/Y coordinates using the Haversine equation on a reference ellipsoid^29,30^.

Unmatched WTs from FAA (DOF and OE-AAA) are considered matching if: (Type 1) two points are within 15.24 meters (50 feet) of each other and have the same online year (p_year), or (Type 2) two points are within 3.05 meters (10 feet) of each other and p_year is within 1 year of each other.

The AWEA WindIQ data are merged into the combined (USGS + LBNL + FAA DOF + FAA OE-AAA) dataset using similar geospatial matching techniques, but with slightly different matching criteria. Because the AWEA data contains WT attribute data, analysts can compare those attributes with USGS and LBNL data to assist in determining and validating matches. Two criteria rules for matching AWEA WTs to the combined dataset are used: (Type 1) two points are within 30.48 meters (100 feet) of each other and have the same hub height (t_hh), rotor diameter (t_rd), and online year (p_year); or (Type 2) two points are within 3.05 meters (10 feet) of each other and p_year is within 1 year of each other. AWEA’s turbine- and project-level attributes (see Table 2) are retained, and non-matching WTs are kept for visual verification.

The distance criteria used in geospatial matching, though somewhat arbitrary, are intentionally restrictive to avoid matching WTs that should not be matched. WT points that are not identified as matches via geospatial matching (but are, in fact, matches) are subsequently identified by analysts via visual verification.

### Visual verification of WT locations

The WTs in the USWTDB are visually verified using high-resolution aerial imagery for four primary reasons: (1) to verify the location of wind WTs and adjust X/Y coordinates to the base of WTs if needed; (2) to identify matches and remove duplicate records from the database; (3) to identify, flag, and remove records where WTs have been dismantled and removed; and (4) to identify, flag, and remove misidentified records where no utility-scale WT actually exists. Records that are not utility-scale WTs may include, for example, small water-pumping windmills, meteorological towers, cell towers, residential-scale WTs, or other structures that do not belong in the USWTDB, but may exist in source datasets.

Visual verification methods closely follow those of Diffendorfer *et al*.^1^. Four aerial imagery sources are used: Digital Globe, Bing Maps Aerial, National Agriculture Imagery Program (NAIP), and Google Earth. Analysts rely primarily on Digital Globe, which typically has the most recent imagery and is available to use as a base layer in ArcGIS with an EnhancedView Web Hosting Service (EV-WHS) account and plug-in. The image source used for verification is recorded in the “t_img_srce” attribute.

The merged and combined dataset is imported into ESRI ArcMap, and a shapefile is generated. A base map image from Digital Globe is added to the ArcMap file, and WTs newly added to the database are examined individually by analysts each quarter. Existing WTs with low location confidence scores (t_conf_loc = 1) are also reexamined quarterly, and those with medium location scores (t_conf_loc = 2) are reexamined at least annually. If Digital Globe imagery does not show a WT, Google Earth and Bing Maps Aerial are checked. For any record where the WT is not seen in any imagery due to recent imagery being not yet available, cloud cover, or other reasons, the WT location confidence (t_conf_loc) is entered as 1 (low). If the location shows partial construction (e.g., WT blades on the ground, only tower installed) or partial cloud cover, or other aspects of the location verification are uncertain, t_conf_loc is entered as 2 (medium). If the imagery clearly shows a fully installed WT, t_conf_loc is entered as 3 (high) for that record.

The map scale used while verifying and digitizing WTs depended on location, need, and imagery availability. Authors used aerial imagery as a layer in ArcMap with a typical assessment map scale of 1:2,000 to 1:5,000; none of the spatial verification correction was done at a map scale smaller than 1:5,000.

In the 2015 USGS dataset, 48,061 WTs had already been visually verified and assigned “high” location confidence scores. These “high confidence” records are not reexamined unless data from FAA, AWEA, or other sources suggest a possible alteration or decommissioning of the site. All of the “low” and “medium” confidence locations from the USGS dataset (*n* = 895) were reexamined between March 2017 and March 2018. Every new (i.e., not matching the preceding USGS nor preceding USWTDB verified data) point added to the database from LBNL, AWEA, DOF, or OE-AAA is also visually verified.

Over time, imagery access has evolved and improved. Digital Globe is now the preferred imagery source (57% of all WTs in the USWTDB; 99% of WTs installed since 2015), but in the 2015 USGS turbine dataset most of the imagery used was from Bing Maps Aerial or NAIP (31% and 12% of WTs in the USWTDB, respectively). In rare cases when no other imagery showed a WT, Google Earth was independently checked outside of ArcMap. Imagery cataloging for Bing Maps Aerial, Google Earth, and the initial older data delivery systems of Digital Globe was not always performed, this is shown as 〈Null〉 in the t_img_date field.

### Wind turbine and project attributes

Characteristics of the WTs and their respective projects are compiled from the five sources shown in Table 1.

The methods to gather WT and project attributes for the USGS turbine dataset were described in Diffendorfer *et al*.^1^. LBNL has been gathering WT and project-level data for over 10 years to analyze and produce the annual Wind Technologies Market Report^31^ and other research projects. The LBNL WT data came from a variety of sources, including: AWEA and FAA data, Energy Information Administration (EIA) data, original equipment manufacturers (OEMs), project developers and owners, and via online searching. AWEA’s data are sourced from the FAA, project developers, owners, and OEMs.

The FAA datasets (DOF and OE-AAA) provide four attributes used in the USWTDB: latitude, longitude, total WT height, and year the WT was built.

The sources of each variable in the USWTDB are outlined in Table 2, and full descriptions of each variable are provided in the “Data Records” section below.

**Fig. 2:**
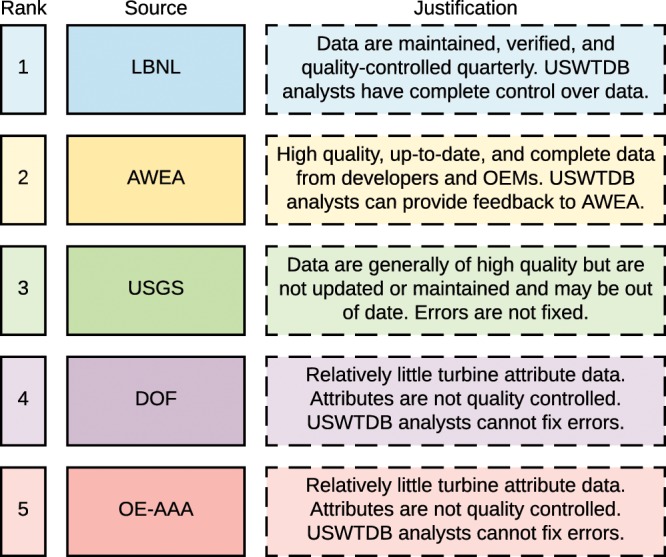
Hierarchy of WT and project attribute source data.

To populate turbine attributes in the USWTDB, we developed a hierarchy across data sources based on the overall quality, timeliness, and accuracy of turbine attributes. In general, we have very high confidence in LBNL data and AWEA data, as these datasets are continuously updated, regularly checked for quality control, and typically corroborate each other (in the case of LBNL data, only *project* level data are regularly updated, as noted above. However, the LBNL project dataset includes information about the turbine manufacturer, model, capacity, hub height, and rotor diameter used in the project). We have slightly less confidence in the 2014 USGS WT data since it is out of date, and we have lower confidence in the accuracy of the FAA data sources since relatively few attribute data are provided and they are not regularly quality controlled. Given the hierarchy outlined in Fig. 2, attribute data are populated in the following order: 1. LBNL data, 2. AWEA data (use if LBNL data are missing), 3. USGS data (use if LBNL and AWEA data are missing), 4. FAA DOF data (use if LBNL, AWEA, and USGS data are missing), and, 5. FAA OE-AAA data (use if all others are missing).

A number of variables (see Table 2) are added to the USWTDB by analysts during visual verification and/or post-processing. To generate t_state, t_county, and t_fips, a spatial join is conducted in ArcMap to join the USWTDB WT points to a shapefile of US states, counties, and Federal Information Processing Standards (FIPS) codes (a five-digit code which uniquely defines US counties and county equivalents) from the US Census Bureau. When not already populated from other data sources, p_name is replaced with the word “unknown” followed by the county in which the WT is located. If p_cap and p_tnum are not populated by USGS or LBNL source datasets, they are calculated by summing the WT capacity in each unique project name. The WT rotor swept area (t_rsa) is calculated using the following equation:

$$t\text{\_}rsa=\pi \ast {\left(\frac{t\text{\_}rd}{2}\right)}^{2}$$

A WT attribute confidence score (t_conf_attr) is assigned to each WT in the USWTDB based on 9 turbine- and project-level attributes (identified in Table 2). A score of 1 indicates “low” confidence; these WTs have only the basic attributes (total height and install year) provided by FAA, but no other attributes populated. A score of 2 indicates “partial” confidence; these WTs either have some (but not all) of the additional six attribute fields populated, or they have all fields populated but the AWEA attribute data conflicts substantially with LBNL data. A “substantial” conflict was defined as any of the following differences: p_year ±4 years; t_hh ±10 meters; t_rd ±10 meters; t_ttlh ±15 meters; t_cap ±250 kW. A t_conf_attr score of 3 indicates the nine attributes are fully populated, and (if applicable) corroborated by multiple data sources.

### Removing duplicates, dismantled, residential-scale, and non-turbine points

Before compiling the final database, analysts remove a number of duplicate or multiple points that were internally flagged as such in the visual verification process. Before removal, their characteristics are cross-referenced and merged together, then collapsed into a single record so that data fields and attributes about a single WT from multiple data sources are retained.

Dismantled WTs are defined as ones that were seen in imagery previously or known to be built and operating at some point in time and have since been verified as dismantled and removed in recent imagery. This may differ from WTs defined as *decommissioned* in other data sources, which can be described as no longer producing power. Only WTs that have been physically removed (and verified as such via imagery) are removed from the USWTDB. Dismantled WTs are retained internally in a separate database for potential future analyses.

Any residential-scale (generally rated less than 65 kW) WTs found in the database are removed. This is done because data for these small WTs were inconsistently included in source data. A filter removes any WTs that are both less than 30 meters in total height, and less than 65 kW in rated capacity. In cases of unknown total height and/or capacity, or if either field’s value exceeded the cut-off, the WT is left in the database and visually examined. Some very old (pre-1990) WTs installed in California are quite small, both in terms of rated capacity and total height, but would still be considered part of utility-scale wind projects (which utilized smaller WT technology at the time), and thus have been purposely retained. Some residential-scale WTs are flagged and removed after visual inspection. Water-pumping windmills, meteorological towers, and other non-WT infrastructure identified via visual inspection are also removed from the public USWTDB.

**Fig. 3:**
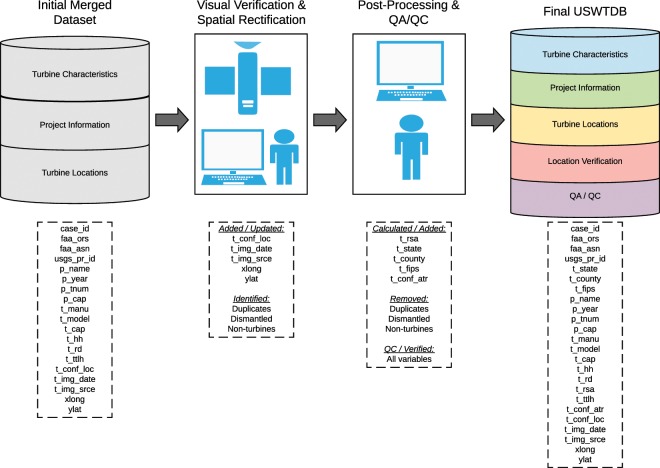
Outline of visual verification, post-processing, and QA/QC processes.

A graphical outline of the visual verification, post-processing, and QA/QC processes used to generate the USWTDB is shown in Fig. 3.

### Quarterly updates

One of the most unique and advantageous aspects of the USWTDB is that it is updated quarterly. Each quarter, new data are gathered from AWEA and FAA, and the steps described above are repeated to add those new WTs to the database, and/or append additional attribute data to existing records. This new data includes newly built WTs as well as retrofits, dismantled WTs (removed from dataset), and updates to existing records. Quality control and technical validation are also conducted each quarter to continually clean and improve the database. A new USWTDB is expected to be released in January, April, July, and October of each year.

## Data Records

The data are distributed as public domain and are available in multiple formats. The most recent quarterly updated database (in Shapefile and CSV format), metadata (XML), and a quarterly changelog (text file) are all housed in the US Geological Survey’s ScienceBase catalog^3^. The data (in Shapefile, GeoJSON, and CSV formats), metadata (in XML format), as well as static and dynamic web services are also available at https://eerscmap.usgs.gov/uswtdb/data/. Data are also available via an application programming interface (API) using a base path of https://eersc.usgs.gov/api/uswtdb/v1/, which enables users to programmatically query data and stay in sync with USWTDB updates.

The USWTDB is updated quarterly, and only the most recent version is available in ScienceBase^3^. Analysts from LBNL store all previous versions of the database in-house; previous versions are available by contacting the corresponding author.

**Fig. 4:**
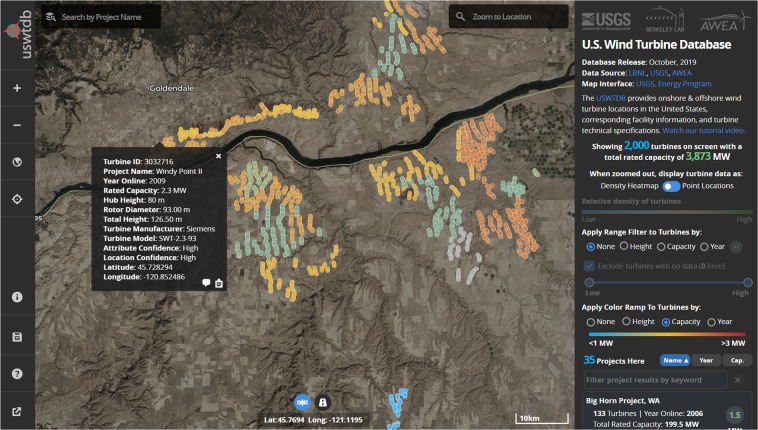
Screen capture from USWTDB web-viewer showing WT locations, attributes, and related information.

An interactive web-based map application hosting the most-recent database version is available (https://eerscmap.usgs.gov/uswtdb/viewer/) and accessible to any user with an internet connection and web browser (see Fig. 4). We used open-source web mapping libraries to create a highly interactive, customized three-dimensional map experience that can be leveraged in both desktop and mobile platforms. Users can interact with the data using a variety of highly dynamic tools like timeline animations, advanced filtering, on-the-fly statistical tools, and dynamic data symbolization.

The dataset contains the fields detailed below.

**case_id**: unique stable identification number.

**faa_ors**: FAA unique identifier for each WT for cross-reference to the FAA digital obstacle files^6^.

**faa_asn**: FAA unique aeronautical study number for cross-reference to the FAA obstruction evaluation airport airspace analysis (OE-AAA)^7^.

**usgs_pr_id**: unique, stable object number for cross-reference to the 2014 USGS Turbine dataset^2^.

**t_state**: state where WT is located.

**t_county**: county where WT is located.

**t_fips**: state and county FIPS code where WT is located, based on spatial join of WT points with US state and county shapefile.

**p_name**: name of the wind power project that the WT is a part of.

- Project names are typically provided to AWEA by the developer; some names are identified via other internet resources, and others are created by the authors to differentiate them from previous projects. Values that were unknown were assigned a name based on the county where the WT is located.

**p_tnum**: number of WTs in the wind power project.

**p_cap**:cumulative capacity of all WTs in the wind project, in Megawatts (MW).

**t_year**: year that the WT became operational and began providing power, which may differ from the year that construction began.

**t_manu**: name of the original equipment manufacturer of the WT.

**t_model**: manufacturer’s model name of the WT.

**t_cap**: rated WT capacity in kilowatts (kW) at the WT’s rated wind speed.

**t_hh**: WT hub height in meters (m).

**t_rd**: WT rotor diameter in meters (m).

**t_rsa**: WT rotor swept area in square meters (m^2^).

**t_ttlh**: WT total height from ground to tip of a blade at its apex in meters (m).

**t_conf_atr**: level of confidence in the WT attributes.

- 1—Low confidence: no attribute data beyond total height and year.
- 2—Partial confidence: incomplete information or substantial conflict between data sources.
- 3—Full confidence: complete information, consistent across multiple data sources.

**t_conf_loc**: level of confidence in WT location.

- 1— Low Confidence: No WT shown in image; image has clouds; imagery older than WT built date.
- 2— Partial confidence: image shows a developed pad with concrete base and/or WT parts on the ground.
- 3— Full confidence: image shows a fully installed WT.

**t_img_date**: date of image used to visually verify WT location. May differ from actual date of image capture – see usage notes below.

**t_img_srce**: source of image used to visually verify WT location.

- Digital Globe - Rapid Delivery of Online Geospatial-Intelligence (RDOG) from a National Geospatial Intelligence Agency contract under a NextView License https://evwhs.digitalglobe.com. Integrated to ESRI ArcMap using the ImageConnect with EV-WHS (for US Government Users) plug-in.
- Bing Maps Aerial – ESRI ArcGIS base maps imagery. Source: ESRI, DigitalGlobe, GeoEye, Earthstar Geographic’s, CNES/Airbus DS, USDA, USGS, AeroGRID, IGN, and the GIS User Community.
- NAIP - National Aerial Imagery Program, USDA Ortho Imagery. http://datagateway.nrcs.usda.gov/.
- Google Earth. http://www.google.com/earth/download/ge/.

**xlong**: longitude of the WT point, in decimal degrees calculated in ArcMap using the calculate geometry tool with the North American 1983 (NAD 83) coordinate system.

**ylat**: latitude of the WT point, in decimal degrees calculated in ArcMap using the calculate geometry tool with the North American 1983 (NAD 83) coordinate system.

## Technical Validation

USWTDB data undergo rigorous QC and validation using several methods. Those include: (1) the use of high-resolution aerial imagery to validate WT locations, (2) the comparison and corroboration across multiple source datasets, (3) extensive quarterly internal review and QC by the authors, (4) quarterly external review by technical experts outside the core database team, (5) internal review of the quarterly data and metadata by non-team members as part of USGS Fundamental Science Practices, and (6) feedback from database users.

**Fig. 5:**
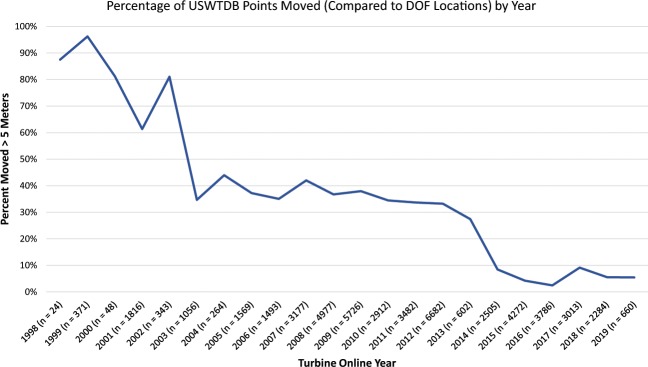
Percentage of WT points moved for locational accuracy, by year (as of October, 2019).

**Fig. 6:**
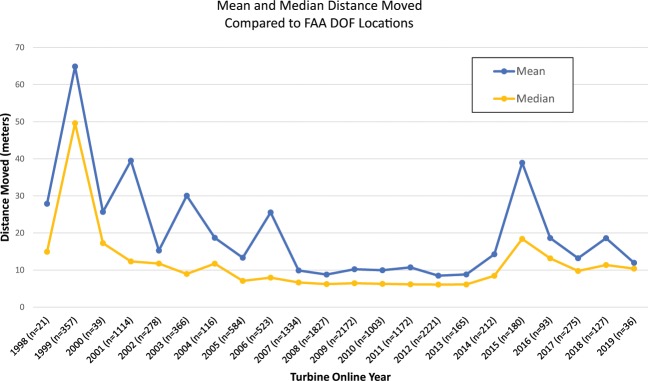
Average distance WT points were moved, by year (as of October, 2019).

**Fig. 7:**
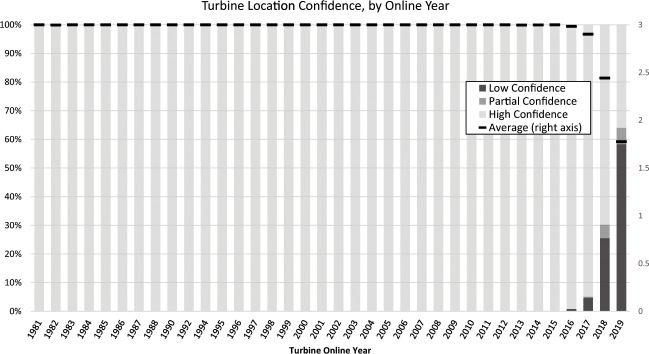
WT location confidence score, by year online (as of October, 2019).

The use of aerial imagery to verify WT locations was described in detail in the methods section. In the time period between March 2017 and October 2019, over 43,500 WT points were visually examined, over 12,500 duplicate points were identified and removed, over 4,900 WTs were marked as dismantled and removed, over 4,100 points were moved for locational accuracy, and over 660 points that were not utility-scale WTs were identified and removed. Human error was minimized in the visual verification process through the use of internal checks and peer-review by multiple team analysts. Figure 5 shows the percentage of WT points installed each year that were moved for locational accuracy, and Fig. 6 shows the average distance WTs were moved by installed year. WT points in the USWTDB are adjusted to the base of the turbine at tolerance of 10 meters at a minimum (i.e., WT points that appear to be within 10 m of the WT base are not moved). As of October 2019, 58,026 (95.8%) of the 60,576 WTs in the USWTDB have the highest location confidence score, while 288 (0.5%) have partial location confidence, and 2,262 (3.7%) have low confidence (see Fig. 7; these scores are defined in the “Data Records” section above). Location confidence is very high overall, but tends to be lower for more recent WTs since available aerial imagery may lag actual installations by several years (this explains the reduced confidence in 2018–19 shown in Fig. 7).

WT attribute data are thoroughly reviewed by corroborating attributes between source datasets and further examining WTs with conflicting data. In such cases, attributes are corroborated with EIA data or by searching the wind project name online to verify characteristics. Text formatting errors, including the spelling of project names, manufacturers, or model names, are found and corrected. Analysts also compare the total height (t_ttlh) to the values for hub height (t_hh) and rotor diameter (t_rd). If total height differs from hub height plus ½ rotor diameter by more than 2 meters, the WT specifications are rechecked and changes are made where data could be improved. Similarly, analysts compare the total project rated capacity (p_cap) to the project number of WTs (p_tnum) and the WT rated capacity (t_cap). In cases where p_cap does not equal p_tnum * t_cap, analysts make changes where data could be improved. These QC reviews are conducted on a quarterly basis.

**Fig. 8:**
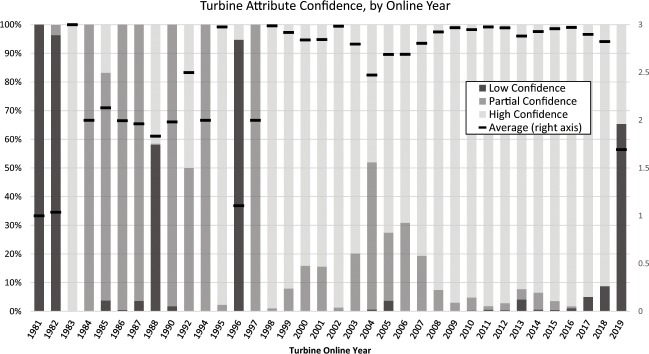
WT attribute confidence score by year online (as of October, 2019).

As of October 2019, 50,545 (83.4%) of the 60,576 WTs in the USWTDB have the highest attribute confidence score, while 5,768 (9.5%) have partial attribute confidence, and 4,263 (7.0%) have low confidence. Attribute confidence tends to be much higher for more recent installations, though it can take a quarter or two between the actual installation of wind projects and when characteristics about them becomes available, as shown by the sharp decline in confidence in 2019 (see Fig. 8).

As part of the USGS Fundamental Science Practices and ensuring the integrity of data products, the data and metadata are reviewed quarterly by individuals outside of the core database development team. This review process incorporates feedback from two technical specialists within the USGS. Their quarterly review logs and comments (along with our responses) can be provided upon request. Not every point is reviewed each quarter, but a sample of newly added turbines is rechecked visually. The metadata was initially reviewed extensively but for subsequent updates, only the changes are examined and the metadata is run through a USGS metadata parser that validates xml records against FGDC’s Content Standard for Digital Geospatial Metadata (CSDGM) standards.

We also receive valuable feedback from users. The original USWTDB was released to the public on April 19, 2018, and an email address was provided for users to submit feedback. We have since received over 55 emails with comments regarding corrections, additions, or suggested features. Many of those comments were written by practitioners (developers, operations staff, etc.) in the wind industry. These comments are validated and incorporated into the database each quarter as they are received. As well, USWTDB analysts conducted a series of formal interviews with key users (including Department of Defense staff, DOE staff, a WT manufacturer, a grid operator, and other researchers) in 2019 to solicit feedback and suggestions. Guided by these formal reviews and feedback channels, the USWTDB continues to evolve and improve.

## Usage Notes

Although the USWTDB is updated regularly, it can only be as up-to-date and accurate as its source data and the verification and release submittal process. In general, the data in the USWTDB lags actual new WT installations by 3 to 6 months. Typically, the WTs appear in FAA data first, and may enter our database as new WTs without any attribute data. In the following quarter, those attributes are infilled after they are populated in the AWEA and/or LBNL datasets.

Users should be aware that ‘null’ data values are represented by −9999 in numerical fields and “missing” in string fields for the shapefile version of the data, though they remain as true nulls in the CSV version. Shapefiles do not support true null values in numeric fields, so we used −9999 to represent null. This value was chosen because we wanted to distinguish null values from 0 values. In the t_img_date field, a date data type 〈nulls〉 were used. These substitute null values should be converted to true nulls before performing any mathematical operations or analyses.

The intent of the USWTDB is to capture utility-scale WTs, and it should not be used as a reference for residential-scale WTs. These small (≤50 kW) WTs are difficult to find in imagery and no comprehensive data source for location or other information about them exists. We made an effort to remove any residential-scale WTs from the database, but a small number may remain if they could not be identified via imagery and/or attribute data.

Beginning in 2017, some WTs that were installed in the mid-2000s have been retrofitted with new rotors and, in some cases, generators and gearboxes. Retrofitting of existing WTs is a new trend but may increase in coming years^31^. We have attempted to capture retrofitted WTs and update their attributes as the retrofits occur, but our updates for retrofitted WTs may lag actual changes by up to one year. Users should be aware that for some WTs, the current attributes (including, e.g., rotor diameter and rated capacity) may differ from the attributes of that WT when it was first installed.

The t_img_date can differ from the actual date the image was captured in some cases. In the case of mosaic images (used by Digital Globe), this is the date of the mosaic creation – not the actual images making up the mosaic. NAIP imagery does not indicate month and day; for those records month and day were set to 01/01.

We have attempted to remove all dismantled WTs from the USWTDB, but this will always be an ongoing effort. We have developed standards to flag and re-examine older, potentially decommissioned WTs, but some WTs may remain in the USWTDB that are, in fact, dismantled. Users should also be aware that we do not remove WTs that are still standing but not producing power. Other data sources may define such WTs as “decommissioned”, but they remain in the USWTDB unless they have been physically dismantled and removed.

Users should bear in mind that although we made great efforts to validate the WTs’ location and attribute data, none of the WTs are field verified. We welcome user feedback to improve the accuracy and quality of the USWTDB at any time.

The USWTDB will continue to evolve and improve in the future. We are currently working to incorporate EIA Plant Codes (project-level IDs) into the database that will enable further cross-validation and utilization with data reported to the EIA and other federal agencies. Finally, we are continuously working to identify and remove dismantled WTs, characterize retrofitted WTs, and otherwise improve the quality and robustness of the USWTDB.

## Data Availability

Several custom scripts were developed to process, manage, and clean the data using Stata 15.0. Two scripts are used to merge the source datasets using full outer join and geospatial methods. Two of the source datasets^4,5^ are not publicly available, but were accessed via the CRADA described above. A third script updates the dataset and removes any duplicates based on the manual entries from visual verification. A fourth script formats and condenses the “master” dataset (which has separate attribute fields from each source dataset and also stores dismantled WTs, non-WT records, and old versions of retrofitted WTs) to compile the cleaned “public” dataset described in this paper. Visual verification of WT locations was conducted manually using ESRI ArcMap. Users may contact the corresponding author for details about these scripts and source data.
